# Effect of Mild Thyrotoxicosis on Performance and Brain Activations in a Working Memory Task

**DOI:** 10.1371/journal.pone.0161552

**Published:** 2016-08-18

**Authors:** Anna Göbel, Marcus Heldmann, Martin Göttlich, Anna-Luise Dirk, Georg Brabant, Thomas F. Münte

**Affiliations:** 1 Department of Neurology, University of Lübeck, 23538, Lübeck, Germany; 2 Department of Internal Medicine I, University of Lübeck, 23538, Lübeck, Germany; Leibniz Institute for Neurobiology, GERMANY

## Abstract

**Aims:**

Disturbed levels of thyroid hormones are associated with neuropsychiatric disorders, including memory impairments. The aim of this study was to evaluate effects of mild induced thyrotoxicosis on working memory and its neural correlates.

**Methods:**

Twenty-nine healthy, male subjects with normal thyroid state participated in the study. Functional MRI was acquired during a working memory task (n-back task) before and after ingesting 250 μg L-thyroxin per day for a period of eight weeks. In addition, neuropsychological tests were performed.

**Results:**

In the hyperthyroid condition the subjects showed slower reaction times, but a higher accuracy in the 0-back version of the memory tasks. Fewer differences between euthyroid and hyperthyroid state were seen for the more difficult conditions of the n-back task. FMRI revealed effects of difficulty in the parahippocampal gyrus, supplementary motor area, prefrontal cortex, anterior cingulate cortex, posterior cerebellum, rolandic operculum and insula (p<0.05, FWE corrected). When comparing euthyroid and hyperthyroid condition in relation to task-induced activation, differences of activation were found in the right prefrontal cortex as well as in the right parahippocampal area. In the psychological assessment, the alerting effect in the Attention Network Task (ANT) and four out of five parameters of the auditory verbal learning test (AVLT) showed an increase from euthyroid to hyperthyroid state.

**Conclusions:**

It can be concluded that even a short-term intake of thyroid hormones leads to an activation of brain areas associated with working memory and to an improvement of accuracy of working memory tasks.

## Introduction

Thyroid hormones are important modulators of cognitive functions [1] during adulthood, whereas thyroid hormone deficiency during brain development can lead to irreversible cognitive and neurological impairments [2,3]. Our hypothesis is, that thyrotoxicosis does not only have an impact on memory and mood, but also on brain function. While cognitive functions can be dramatically impaired in hypothyroidism to the extent that they mimic dementia [4–14], some deficits are also present in thyrotoxicosis [3,15,16]. Typical symptoms in hyperthyroidism include reduced concentration and memory performance [2] and a recent review by Samuels et al. [17] pointed out that “subtle deficits in specific cognitive domains (primarily working memory and executive function) likely exist in subclinical hypothyroidism and thyrotoxicosis”. Studies show that the middle/inferior frontal gyri, prefrontal cortex, premotor areas, supplementary motor area, anterior cingulate cortex and parietal areas are activated by the n-back task. In hypothyroidism, these areas are not all activated, only in the parietal areas and premotor areas, the other areas could not be activated any more in thyrotoxicosis. It can therefore be suggested that working memory function cannot be performed in these areas during hypothyroidism [18]. For hyperthyroidism and thyrotoxicosis the cerebral processes behind the endocrine-induced working memory changes are yet unclear. The first study showing the effects of hyperthyroidism on cerebral activity described an increased cerebral blood flow but normal oxygen consumption [19]. Thyrotoxicosis correlated psychic symptom are correlated to metabolic changes in the limbic, paralimbic system, posterior cingulate cortex and temporal lobe, as addressed in a PET study by M. Schreckenberger et al. [20]. We are therefore one of the first groups to study the effect of hyperthyroidism on brain function.

In the present study we therefore sought to analyze the effect of exogenously administered thyroid hormones on working memory using the n-back task [21] in conjunction with functional magnetic resonance imaging (fMRI). We were motivated by results in the literature that are not quite clear whether thyrotoxicosis leads to an improved [22,23] or reduced [3,15,16] memory ability, or did not have an influence on it [24] and we therefore concluded that further research is neccessary. Extensive research in imaging genetics has attested to the usefulness of the n-back task to define subtle cognitive abnormalities and prefrontal dysfunction. Imaging genetics uses genetic information and fMRI in the same subjects and links neuro-mechanisms to genetic variation. It can therefore show, how individual differences lead to differences in the brain and for example its working memory. In imaging genetics the use of fMRI has been driven by “the intermediate phenotype concept” that posits that neural responses might be more sensitive than overt behaviors and that by neuroimmaging quantitative, mechanistic aspects of altered brain functions can be studied [25]. In the n-back paradigm, subjects are required to monitor a series of stimuli appearing one after the other on a video monitor and to respond whenever a stimulus is presented that is the same as the one presented n trials previously [26–29]. By varying n memory demands can be manipulated [30]. Besides a 1-back and a 2-back condition, we also introduced a so-called 0-back condition, which required subjects to respond to a prespecified target letter, to serve as a baseline condition. In addition to the n-back fMRI-task, participants were also subjected to a comprehensive neuropsychological battery to cover a wide range of cognitive domains. Compared to traditional behavioral data analysis methods, we also included fMRI because of ist objectivity and fMRI enables an understanding of the relationship between activity in certain brain areas and specific mental functions.

## Materials and Methods

### Ethics Statement

The ethical committee of the University of Lübeck had approved all procedures and all subjects gave their written informed consent prior to participation. The study was performed in agreement with the Declaration of Helsinki.

### Subjects

Data was acquired at the University of Lübeck. Twenty-nine healthy, male, right-handed subjects (age range 21 to 49 years, median 30 years, SD 7.33) were recruited for this study. One participant was excluded due to extensive bodybuilding and the off-label intake of steroid hormones. Subjects were screened for general health by physical examination and history taking, for medication and drug abuse by history taking, for thyroid status by laboratory testing and for mood or cognitive disorders by psychological examination. All subjects included in the study had normal structural images as determined by a neuroradiologist showing no signs of atrophy of the cerebral cortex and subcortical structures. All subjects had normal thyroid hormone levels prior to the study (table 1).

**Table 1:** Clinical, epidemiological and psychological characteristics of participants. Means and standard error of the mean are stated. T values are reported for psychological test results under the assumption hyperthyroid > euthyroid state. Significant results are accentuated in bold. RT = reaction time, SD = standard deviation. n.s. = not significant. figure1-wm-goebel (1).tif.

	Euthyroid state mean	Hyperthyroid state mean	T	P
Median TSH level	2.21 (SD 1.27, n = 21)	0.02 (SD 0.02, n = 24)		**<0.001**
Median fT3 level	4.71 (SD 0.89, n = 21)	7.44 (SD 2.49, n = 24)		**<0.001**
Median fT4 level	15.78 (SD 2.51, n = 21)	31.33 (SD 8.17, n = 24)		**<0.001**
Systolic blood pressure mmHg	120.9 (SD 4.5)	124.5 (SD 7.8)		n.s.
Diastolic blood pressure mmHg	71.6 (SD 6.5)	71.4 (SD 5.4)		n.s.
Heart beats per min	68.8 (SD 4.5)	74.8 (SD 7.5)		**0.016**
ANT alerting effect (ms)	27.9 (SD 17.9)	40.2 (SD 16.6)	3.8	**<0.001**
ANT orienting effect (ms)	4.2 (SD 13.6)	4.1 (SD 11.6)	-0.03	n.s.
ANT executive effect (ms)	87.3 (SD 29.6)	80.8 (SD 12.9)	-1.2	n.s.
TAP RT Working memory task (ms)	626 (SD 119)	637 (SD 124)	0.74	n.s.
TAP RT task switching (ms)	429 (SD 79)	454 (SD 81)	1.91	**0.06**
TAP RT divided attention auditive (ms)	546 (SD 89)	545 (SD 76)	-0.04	n.s.
TAP RT divided attention visual (ms)	736 (SD 78)	730 (SD 101)	-0.39	n.s.
TAP RT GoNogo (ms)	522 (SD 54)	528 (SD 65)	0.54	n.s.
TAP N correct Working memory task	13.6 (SD 2.2)	14.0 (SD 1.2)	0.77	n.s.
TAP N correct task switching	17.3 (SD 3.3)	17.9 (SD 0.2)	1.05	n.s.
TAP N correct dual task auditive	15.6 (SD 0.5)	15.25 (SD 1.2)	-1.43	n.s.
TAP N correct dual task visual	16.4 (SD 1.4)	16.68 (SD 0.7)	1.36	n.s.
TAP N correct GoNogo	22.7 (SD 4.1)	23.9 (SD 0.4)	1.59	n.s.
TMT A (s)	20.6 (SD 5.8)	18.4 (SD 5.3)	-1.6	n.s.
TMT B (s)	48.4 (SD 21.2)	52.7 (SD 29.1)	0.76	n.s.
VLMT supraspan	8.26 (SD 1.9)	9.04 (SD 2.3)	1.72	0.09
VLMT learning ability	12.44 (SD 2.6)	13.67 (SD 1.7)	3.51	**0.001**
VLMT global learning rate	54.59 (SD 10.8)	59.52 (SD 8.4)	2.6	**0.014**
VLMT list interference	0.04 (SD 1.09)	1.12 (SD 1.07)	4.07	**<0.001**
VLMT temporal interference	0.04 (SD 1.2)	1.58 (SD 2.3)	3.11	**0.004**
BDI score	1.74 (SD 2.3)	2.66 (SD 3.1)	1.8	n.s.
SCL-90R GSI	0.17 (SD 0.21)	0.16 (SD 0.2)	-0.23	n.s.
SCL-90R PSDI	1.07 (SD 0.37)	0.97 (SD 0.26)	-1.6	n.s.
SCL-90R PST	13.22 (SD 13.5)	13.15 (SD 14.2)	-0.05	n.s.
ASTS Sadness	3.44 (SD 1.2)	3.52 (SD 1.4)	0.2	n.s.
ASTS Hopelessness	3.15 (SD 0.4)	3.41 (SD 1.5)	0.9	n.s.
ASTS Tiredness	9.56 (SD 4.7)	10.78 (SD 5.1)	1.3	n.s.
ASTS Anger	3.3 (SD 0.8)	3.22 (SD 1.15)	-0.26	n.s.
ASTS Positive mood	17.22 (SD 4.8)	18.69 (SD 4.52)	1.2	n.s.

### Experimental Design

Two identical experimental sessions were conducted prior to and after administration of 250 μg L-thyroxine per day for 8 weeks. Between the two sessions, no medication other than L-thyroxine was allowed. In both scanning sessions, subjects performed the n-back task in a block design with three conditions. In the 0-back condition subjects had to press a button whenever they saw a prespecified target letter. This served as a baseline condition. In the 1-back condition a reaction was required whenever the current letter was identical to the immediately preceding letter. In the 2-back condition participants had to press a button whenever the letter was identical to the one seen two letters before. The letters were displayed for 600 ms with an inter-trial interval of 800 ms. Each block comprised 22 stimuli including 3 targets. Prior to each block the condition (0-back, 1-back, 2-back) was defined by an instruction cue that was displayed for 2 seconds. Four blocks of each condition were presented.

### Standardized Symptom Rating Scale for Assessing Hyperthyroidism Related Symptoms

All subjects filled out the standardized “symptom rating scale” [31,32] which tests for hyperthyroidism-related symptoms such as anxiety, sweating, bodily discomfort, restlessness, tremor, hunger, palpitation, concentration, tiredness, appetite, heat and activity. Scores of +20 or more are considered suggestive of hyperthyroidism.

### MRI Data Acquisition and Analysis

Imaging data were collected on a 3.0 T Philips Achieva MR-scanner (Philips Healthcare, the Netherlands) located at the University Hospital Lübeck. High resolution structural images were obtained applying a T1-weighted 3D turbo gradient-echo sequence with SENSE (image matrix 240×240; 180 slices; 1×1×1 mm3 spatial resolution). Functional MRI data were recorded using a T2*-weighted single-shot gradient-echo EPI sequence applying the following parameters: repetition time TR = 2000 ms; echo time TE = 29 ms; isotropic 3 mm voxel size; field of view 192 mm; 40 slices, flip angle 80° and SENSE factor R = 1.8.

Preprocessing was performed using the SPM8 software package (http://www.fil.ion.ucl.ac.uk/spm/). The first 4 images of each dataset were removed in order to allow the subjects to adjust to the environment and for magnetization equilibrium. To improve inter-subject registration, we applied Diffeomorphic Anatomical Registration Through Exponentiated Lie Algebra (DARTEL) [33]. Data processing steps were performed according to Ashburner (2010) (http://www.fil.ion.ucl.ac.uk/~john/misc/VBMclass10.pdf). At first, the functional images were phase-shifted with reference to the middle slice in order to correct differences in slice acquisition time. In the next step, the images were realigned to remove movement artifacts. After realignment, the images were normalized to the MNI-T1 template. This was performed by matching gray and white matter to a reference [34]. Afterwards, the normalized images were smoothed with a Gaussian kernel of 8 mm full-width half-maximum. No subjects had to be excluded because of head movement, since the displacements of the realignment parameters with respect to the first image of the series were all smaller than cutoff-criterion of 3 mm and the rotations smaller than 3 degrees. There were no differences in movement between the first and second session.

Functional brain images were assessed as follows: The group comparison of euthyroid versus hyperthyroid state was analyzed by a statistical design with the 1-way analysis of variance test (ANOVA) in functional MR. Statistical images were assessed for voxel-wise significance correcting for Family-wise error (FWE; q = 0.05) for comparing the difficulty of n-back test conditions. The difficulty of the n-back task condition is the difference in difficulty of 0-back (= control task), 1-back (= easier task) and 2-back (= harder task) condition. For the other analyses significance was set at an uncorrected threshold of p<0.005. If not stated otherwise, we report the mean and standard deviation of the data.

### Psychological Assessment

A comprehensive psychological assessment battery was used to assess changes in different cognitive domains. The test results were corrected for multiple comparison (Bonferroni correction). The *Attention Network Task (ANT)* assesses a number of attention-relevant measures within a single task [38,39]. By introducing different kinds of cues and flanker stimuli, so called “attention network efficiencies” can be calculated. We assessed “alerting”, “orienting”, and spatial cueing effects.

A number of further computerized tests of attention were taken from the “Testbatterie zur Aufmerksamkeitsprüfung” (battery for attention testing) [35], a standardized battery widely used in Europe. The Go/Nogo-test assesses the ability to react to specified target stimuli and—at the same time—to withhold reactions to similar non-target (nogo) stimuli. The divided attention task assesses the ability to allocate attention to an auditory and a visual stimulus series. The Task Switching test (in German “*Aufgabenwechsel*”) estimates the ability to flexibly change between different stimulus-response mappings and thus assesses the quality of executive control functions. Finally, an n-back working memory task (Test “*Arbeitsgedä*chtnis”) was also used.

The Trail-Making-Test (TMT) is a paper and pencil test which assesses visuomotor tracking (part A) and conceptual tracking and executive control (part B) [15]. The time to complete part A and B is scored.

The *Verbaler Lern-und-Merkfähigkeits-Test* (VLMT) is the German version of the auditory verbal learning test (AVLT, [16]) and assesses serial verbal list-learning with intermittent distraction, delayed recall and recognition [36]. It allows to compute different parameters characterizing declarative verbal memory such as supra-span, learning rate, long-term encoding and retrieval as well as recognition.

To assess effects on mood and psychological well-being, the Beck Depression Inventory II (German version [37,38]), the Symptom-Check-List SCL 90R [39] and the “Aktuelle Stimmungsskala” (ASTS [40], which is the German version of the “profile of mood state” scale (POMS), were administered.

## Results

The treatment resulted in mild thyrotoxicosis as evidenced by the hormone levels (table 1). In spite of this, the standardized “symptom rating scale” [31,32] did not reveal significant clinical symptoms. There was a tendency towards an increase of systolic but not diastolic blood pressure. Also, there was an increase in heart rate. Of the psychological tests, changes were only seen for the alerting effect of the ANT-task as well as for several parameters of the verbal learning and memory test. A significant alerting effect (mean result in euthyroid state 27.9 ms (SD 17.9), mean result in hyperthyroid state 40.2 ms (SD 16.6), T 3.8, p<0.001), but no significant orienting and executive effect could be spotted in the ANT task. The hyperthyroid state therefore decreased the subjects attention significantly (see table 1).

The other performed psychological tests (TAP task, TMT, BDI, SCL-90R, ASTS) showed no significant differences between subjects in euthyroid and hyperthyroid state (see table 1).

The reaction time for hits in the n-back task was scored (table 2). While there was a difference between euthyroid and hyperthyroid state for the 0-back task (paired student t-test, p = 0.01), no differences were seen for the 1-back (p = 0.418) and 2-back task (p = 0.992).

**Table 2:** Reaction times and performance accuracy of n-back task. Subjects in hyperthyroid state needed a significant longer time in order to conduct 0-back task (paired student t-test p = 0.01), no significant changes for 1-back and 2-back task. Subjects in hyperthyroid state showed a significant higher accuracy in conducting 0-back task as compared to euthyroid state (paired student t-test p = 0.006). No significant changes for 1-back and 2-back task. n.s. = not significant.

Task condition		0-back		1-back		2-back	
Thyroid state		Euthyroid	Hyperthyroid	Euthyroid	Hyperthyroid	Euthyroid	Hyperthyroid
**Reaction time**	**n**	24	24	19	19	21	21
	**Mean**	0.43	0.45	0.49	0.50	0.53	0.53
	**SD**	0.05	0.04	0.05	0.06	0.07	0.07
	**T**	-2.81		-0.83		0.01	
	**df**	23		18		20	
	**p**	**0.01**		n.s.		n.s.	
**Number of correct answers**	**n**	24	24	19	19	21	21
	**Mean**	11.71	14.92	11.68	12.42	8.48	8.81
	**SD**	5.12	2.32	3.07	3.52	3.25	3.49
	**T**	-3.06		-0.78		-0.43	
	**df**	23		18		20	
	**p**	**0.006**		n.s.		n.s.	

The performance accuracy refers to the percentage of correctly reported letters of the total number of letters in the n-back task. Table 2 shows that the performance accuracy was significantly better in the 0-back task in hyperthyroid state as compared to euthyroid state (paired student t-test, p = 0.006). In the 1-back and 2-back task no significant differences could be shown (paired student t-test for 1-back task p = 0.444 and for 2-back task p = 0.674).

**Fig 1:**
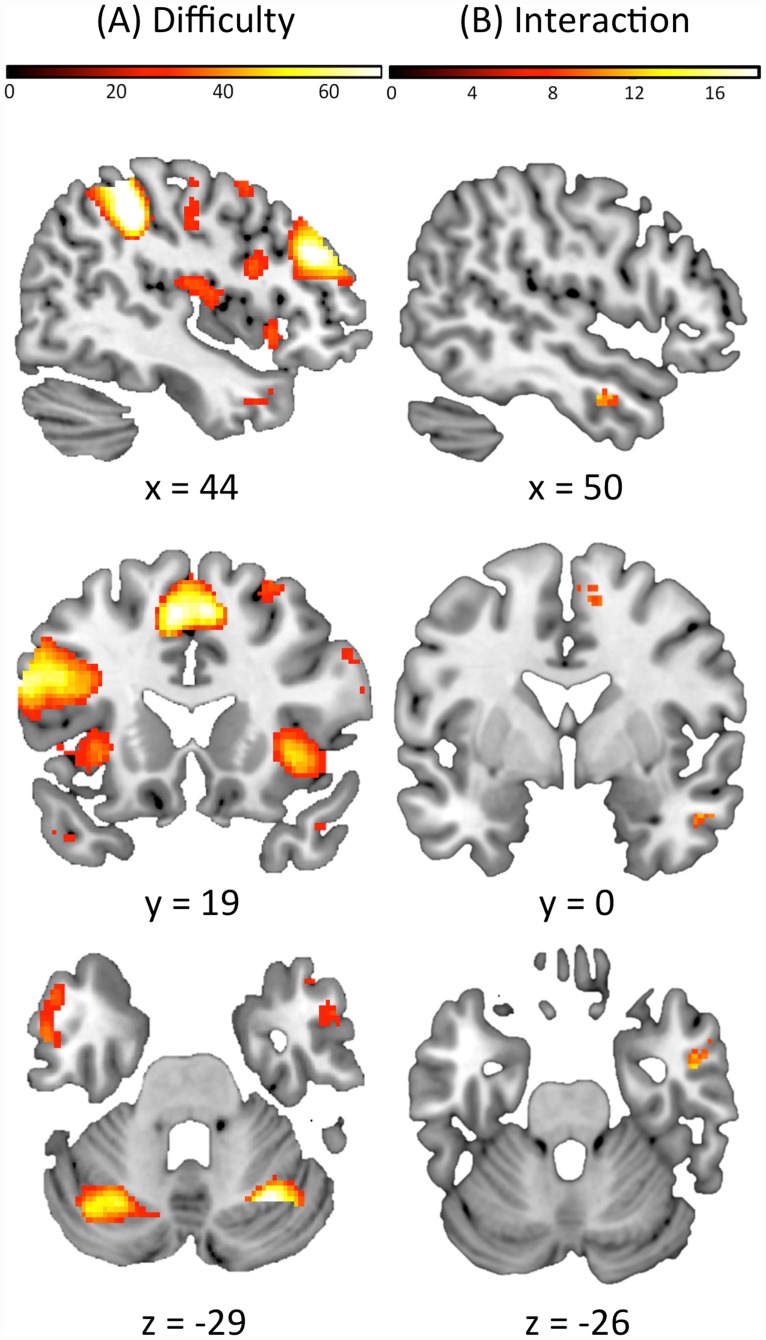
**(A) Regions of BOLD signal intensity in the whole brain for effect of difficulty level in n-back task.** Results for whole brain analysis for all subjects (n = 28), threshold at p<0.05 corrected for FWE. BOLD signal intensity attenuation especially in parahippocampal gyrus, supplementary motor area, dorsolateral prefrontal cortex, rolandic operculum, bilateral anterior cingulate cortex and posteriorcerebellum. The difficulty of the n-back task condition is the difference in difficulty of 0-back (= control task), 1-back (= easier task) and 2-back (= harder task) condition. **(B) Difficuly level in n-back task and thyroid hormone intake interaction.** Results for whole brain analysis with one-way ANOVA in all subjects (n = 28). A cluster in the right parahippocampal region and in the right prefrontal cortex survived the statistical threshold at p<0.005, uncorrected.

With regard to functional MRI a robust modulation of the BOLD activity (see table 3) was found as a function of the different conditions of the n-back task in prefrontal areas (bilaterally), the insula (bilaterally), the parahippocampal region (bilaterally), the posterior cerebellum and the anterior cingulate cortex (p<0.05 FWE corrected at the voxel-level; Fig 1a). An interaction between n-back difficulty and thyroid status was found in the right dorsolateral prefrontal cortex as well as the right parahippocampal area (p<0.005, uncorrected; Fig 1b). Please not that the interaction effect did not survive FWE correction at p<0.05. It is reported here for descriptive purposes.

**Table 3:** Coordinates of MNI space for Regions of BOLD signal intensity in the whole brain for effect of difficulty level in n-back task, difficulty x thyroid status interaction. L = left hemisphere, R = right hemisphere, size = number of functional voxels., BA = Brodmann area.

	Region	Side	BA	x	y	z	Size
Difficulty in n-back task	Parietal Lobe	R	7, 39	32	-46	44	114
	Inferior parietal lobe	R	40	44	-38	46	100
	Precuneus	R	7	12	-68	54	87
	Parietal Inferior Lobe	L	40	-38	-46	48	113
	Parietal Inferior Lobe	L	40	-36	-44	40	99
	Parietal Superior Lobe	L	7	-26	-62	52	86
	Middle Frontal Gyrus	R	10	30	8	56	98
	Precentral	L	6	-30	0	62	93
	Frontal Inferior Triangularisangularis	L	46	-46	28	28	77
	Frontal Inferior Triangularisangularis	L	46	-48	20	26	65
	Frontal Inferior Triangularisangularis	R	46	42	36	26	83
	Middle Frontal Gyrus	R	10	38	52	16	55
	Supplementary Motor Area	L	32	-6	22	44	79
	Supplementary Motor Area	R	32	4	22	46	70
	Supplementary Motor Area	L	32	-6	14	52	68
	Cerebellum Posterior Lobe 6	R		30	-64	-28	76
	Cerebellum Crus 1 Posterior Lobe	R		10	-74	-26	66
	Cerebellum Crus 1 Posterior Lobe	L		-32	-64	-30	62
	Superior frontal gyrus	L	9	-12	50	40	66
	Medial Frontal Gyrus	L	46	-6	56	22	60
	Superior Frontal Gyrus	L	9	-12	58	30	51
	Cingulum Middle	L	24	-4	-8	48	59
	Cingulum Middle	L	32	-8	-28	46	37
	Precuneus	L	7	-6	-52	10	58
	Posterior Cingulum	L	23	-8	-50	28	51
	Posterior Cingulate	L	23	2	-50	18	39
	Middle Frontal Gyrus	L	46	-34	56	16	57
	Postcentral	L	2	-42	-18	52	56
	Postcentral	L	3	-56	-16	46	39
	Postcentral	L	2	-22	-30	64	37
	Insula	R	16	34	22	0	56
	Temporal Middle Lobe	L	21	-58	-6	-18	55
	Temporal Middle Lobe	L	20	-52	-2	-30	44
	Temporal Middle Pole	L	21	-46	8	-34	40
	Frontal Inferior Triangularisangularis	L		-46	-20	16	45
	Rolandic Operculum	L	13	-36	-26	18	44
	Rolandic Operculum	L	22	-50	-6	4	43
	Frontal Superior Gyrus	R	9	16	40	50	43
	Insula	L	16	-30	22	0	43
	Frontal Inferior Triangularisangularis	L	45	-46	16	4	27
	Temporal Superior	R	22	60	-4	4	42
	Rolandic Operculum	R	41, 42	46	-14	16	38
	Rolandic Operculum	R	41, 42	58	-16	14	37
	Frontal Inferior Operculum	R	42	48	10	22	42
	Frontal Inferior Operculum	R	42	56	14	30	33
	Frontal Inferior Operculum	R	42	56	14	20	30
	Angular	L	39	-52	-66	28	35
	Precentral	R	6	46	-14	46	34
	Precentral	R	4	48	-12	56	31
	Middle Temporal Gyrus	R	21	54	6	-28	33
	Middle Temporal Pole	R	21	46	12	-32	33
	Middle Temporal	R	21	48	-4	-30	29
	Precentral	R	4	26	-28	70	32
	Middle Temporal	R	21	56	0	-18	32
	Temporal Lobe	L	21	60	-6	-14	30
	Capsula Interna Extra Nuclear	R	41	-18	4	14	31
	Superior Parietal	L	7	-20	-44	74	31
	Cuneus	R	19	16	-84	34	30
	Superior Occipital	L	18	-14	-94	22	30
	Postcentral	L	2	-58	-6	28	29
	Hippocampus	L	28	-28	-16	-22	29
	Temporal superior Pole	R	22	44	18	-28	27
	Supra Marginal	R	40	50	-30	26	27
	Precentral	R	4	56	-12	44	27
	Temporal Superior Pole	R	38	42	20	-30	26
Interaction n-back task and thyroid hormone status	Parahippocampal	L	21	50	-4	-26	29
	Parahippocampal	L	21	56	4	-24	2
	Supplementary Motor Area	R	6	10	2	54	20
	Temporal Superior Lobe	L	38	-62	-40	16	4
	Temporal Lobe	L	38	42	6	-30	3
	Precuneus	R	7	18	-58	42	3
	Frontal Lobe	L	10	20	30	2	2
	Capsula interna Extra Nuclear	L	41	22	-4	16	1
	Parahippocampal Gyrus Limbic Lobe	R	21	-8	-44	0	2
	Supplementary Motor Area	R	6	4	0	60	1
	Anterior Cingulum	L	24	0	26	-6	1

## Discussion

Our intervention resulted in mild thyrotoxicosis as evidenced by the hormone levels. There were some mild effects on behavior in the working memory task, the alerting measure of the attention network task and the verbal learning and memory test but no clinical signs of hyperthyroidism. The fMRI task showed clear effects of task difficulty and in addition modulations of these difficulty effects by thyroid status.

### Difficulty Effects

A large meta-analysis of fMRI studies employing the n-back task [41] has shown a highly consistent pattern of activation of the lateral premotor cortex, dorsal cingulate and medial premotor cortex, dorsolateral and ventrolateral prefrontal cortex, frontal poles and medial and lateral posterior parietal cortex and medial cerebellum. Our study largely agrees with these results showing activity in the dorsolateral prefrontal cortex, supplementary motor area, parahippocampal area, rolandic operculum, bilateral anterior cingulate cortex and posteriorcerebellum.

Increased difficulty of the working memory task was associated by increased activity in the right dorsolateral prefrontal cortex as well as the right parahippocampal area. This finding is in accordance with the literature, which consistently reported dorsolateral prefrontal cortex activity to scale with difficulty [41–44]. The prefrontal cortical signal decreased at the highest working memory load, coincident with a significant decrement in performance accuracy [42]. The dorsolateral prefrontal cortex is associated with reorganization of materials into familiar or regular structures that is an important component of working memory tasks [41,45]. A significant decrement in performance accuracy can also be seen in this study, in both euthyroid and hyperthyroid state when comparing the accuracy in the 2-back task with the accuracy in the 0-back task (p<0.001).

The parahippocampal region plays an important role in the encoding and recognition of information. Miao et al. [8] showed an increased BOLD signal in the parahippocampal region, when comparing hyperthyroid patients with euthyroid controls. The group analyzed cerebral metabolic changes in Graves disease before and after antithyroid treatment and patients with previously untreated hyperthyroidism showed less activity in the parahippocampus. Treatment with methimazole specifically increased regional activity in the left parahippocampus and the right superior frontal gyrus.

The dorsolateral prefrontal cortex is highly interconnected with many regions of the brain [46]. It is widely believed that the dorsolateral prefrontal cortex serves as a store for short-term memory [26,47–53]. The dorsolateral prefrontal cortex contains most of the dopamine-sensitive neurons that are associated with short-term memory tasks in the cerebral cortex [54,55]. The supplementary motor area is a part of the primate cerebral cortex that contributes to the control of movement, but activity in the supplementary motor area is also related to learning and performance during sequential tasks [56]. The insula and the rolandic operculum link information from diverse functional systems [57]. The cerebellum is a brain region that has long been considered to mainly function in the control of movement [58]. However it is now known, that it is also involved in working memory tasks and that damage to the posterior cerebellum leads to cognitive deficits [59,60]. The anterior cingulate cortex is also required to successfully accomplish the n-back working memory task [61,62]. All of the regions showing an increased BOLD signal in our study are hence linked to cognition and our study therefore is concordant with the current literature. Working memory plays a major part in higher cortical functions like intelligence [42,64], therefore it is important to match the subjects according to their academic background. In this study all subjects therefore had the same level of education.

### Effects of L-Thyroxin

To our knowledge there have been only three further studies analyzing the effect of experimentally raised thyroid hormones on cognitive performance. The first study of Kathmann et al. [65] (i) shows no significant change in memory, attention or visuomotor coordination, but a trend towards an improved verbal fluency after the administration of 100 μg L-thyroxin per day for 3 days to 14 young euthyroid healthy men (20–37 years old). (ii) In the study of Baethge et al. [66] the administration of 500 μg/d for 45 days on average to 11 subjects (4 men, 7 women, mean age 37 years old) did neither change attention, memory, visuospatial organization, verbal learning or verbal fluency. Both of these studies share the disadvantage of a small subject numbers and it is likely that no effect could be seen due to a relatively short period of time in which the thyroid hormones were ingested. The third study of Münte et al. [67](iii) evaluated the effect of experimentally raised thyroid hormone level (ingestion of 300 μg L-thyroxin per day for 3 weeks) on cognitive performance in otherwise euthyroid subject. It was able to detect an improved visual processing in hyperthyroid patients in a placebo-controlled cross-over study in 24 healthy men. Our study evaluates the intake of thyroid hormones with both a larger subject number (n = 28) and a longer duration of intake of the thyroid hormones (8 weeks). It is therefore able to deliver new insights in the question of how hyperthyroidism can influence working memory.

The most common cognitive deficits in patients suffering from hyperthyroidism include poorer performance on tests of attention, working memory, mental alertness and visuomotor speed [36]. In the available studies [23,68] no effect can be shown in working memory. Our study shows a deterioration of the alerting effect in the ANT (p<0.001). The literature approves, since mild deficits of attention, memory and problem solving can be seen in hyperthyroidism [67,69]. It is likely, that the intake of thyroid hormone for only a short-time period may not have such an influence [23,68]. In the auditory verbal learning test (AVLT) a significant improvement when comparing euthyroid to hyperthyroid state could be seen in learning ability (p = 0.001), global learning rate (p = 0.014), interference tasks (p<0.001) and temporal interference (p = 0.004). In short-term hyperthyroidism, AVLT test scores have never been studied, to our knowledge. The improvement in the AVLT after short-term hyperthyroidism may be explained by positive effects resultant to the increased metabolism due to the hyperthyroid state. In subclinical hyperthyroidism no statistical effect could be seen in the literature [70]. It may therefore be that long-term hyperthyroidism does not improve auditory and visual learning because of distraction due to side-effects [71] from the hyperthyroid state and only short-term hyperthyroidism leads to this positive effect. It therefore cannot be concluded that externally administered thyroid hormones lead to “cognitive deficits”–on the contrary. The question remains, whether the cognitive deficits that long-term hyperthyroid patients experience are a result to their hyperthyroid state—it can be argued that a short-term hyperthyroid state may improve cognitive functions [65–67], while a long-term hyperthyroid state for years—besides to other diseases that are linked to hyperthyroidism [71]–will eventually lead to cognitive deficits.

After the intake of thyroid hormones, the subjects increased their 0-back task accuracy significantly (p = 0.006), but required a longer reaction time (p = 0.01). It seems that the intake of thyroid hormones only plays a role in the less difficult n-back task (0-back task as compared to the 1-back and 2-back task). To our knowledge, the effect of short-term intake of thyroid hormones on n-back task performance has never been studied. Significant disturbances of working memory and n-back task performance in both reaction time and number of correct answers have been found in hyperthyroid patients suffering from Graves’ disease in comparison to healthy subjects [68]. Longer disease duration was associated with worse n-back task results [68]. Our study analyzed short-term intake of thyroid hormones, which resulted in an expectantly less intense reduction of n-back task performance. In the hyperthyroid state a significantly increased accuracy in the 0-back task can be shown. This can be explained by positive effects resultant to the increased metabolism due to the hyperthyroid state. These positive effects decrease, the more difficult the n-back task gets. This may be explained by the rising distraction due to side-effects [71] in the hyperthyroid state. It is likely that these side effects also lead to a significantly slower reaction time in the 0-back task. Additionally, hyperthyroidism is often accompanied by impaired concentration or emotional instability [36], which could also explain the increased reaction time. It is possible that a short-term effect of thyroid hormones therefore increase the correctness in 0-back task, but does not affect the more difficult 1-back and 2-back tasks. A long-term effect of hyperthyroidism like it is present in Graves ‘ disease on the other hand may lead to impaired working memory test results at all difficulty levels.

### Limitations to Our Study

One problem of this study is that it was conducted in an open non-balanced fashion. Thus, any changes in brain activation and behavior from session 1 to session 2 could, in principle, also be due to an order effect. However, the literature [72] suggests that the n-back task is well suited for both within-, as well as between-subject designs, including pharmaco-fMRI. Also, the presentation of the n-back task was performed randomly and by this we decreased the possibility of subjects recalling the targets. According to the study group, this study design seems to provide task-specific fMRI reliability performance measures that can show the optimal use, powering and design of fMRI studies.

Another limitation to our study is that to some extent we relied on reverse inference [73,74] in the interpretation of the activation patterns. Because the n-back task has been explored by fMRI by dozens of studies and, moreover, a number of meta-analyses have been published [41,75,76] the response pattern in this task is very clear. In addition, Hutzler [77] recently has rehabilitated reverse inference by arguing “that reverse inference cannot be disregarded as a fallacy per se. Rather, the predictive power of reverse inference can even be "decisive"-dependent on the cognitive process of interest, the specific brain region activated, and the task-setting used.”

With regard to our manipulation, it is unclear whether a longer time period of L-thyroxin intake would result in greater effects. Moreover, the dose of 250 μg per day is probably at the lower end of what is required to induce clinically relevant thyrotoxicosis.

## Conclusion

Our results show an activation of brain areas associated with working memory, including the parahippocampal gyrus, supplementary motor area, prefrontal cortex, anterior cingulate cortex, posteriorcerebellum, rolandic operculum and insula. The intake of thyroid hormones increased BOLD activity in the right prefrontal cortex as well as the right parahippocampal area. An increased BOLD activity in the hyperthyroid state, as compared with the euthyroid state, can be seen while performing the 2-back task in the the right supplementary motor area, the right dorsolateral prefrontal cortex and the left rolandic operculum, as well as the 1-back and the 2-back task in the right parahippocampal gyrus. All stated areas are associated with memory processes. Our data shows that short-term experimentally induced hyperthyroidism leads to an improvement in accuracy of working-memory tasks. In psychological assessment, the alerting effect in the Attention Network Task (ANT) and four out of five parameters of the auditory verbal learning test (AVLT) showed an increase from euthyroid to hyperthyroid state. It therefore can be concluded that even a short-term intake of thyroid hormones lead to an activation of brain areas associated with working memory.
